# International Network of Chronic Kidney Disease cohort studies (iNET-CKD): a global network of chronic kidney disease cohorts

**DOI:** 10.1186/s12882-016-0335-2

**Published:** 2016-09-02

**Authors:** Thomas Dienemann, Naohiko Fujii, Paula Orlandi, Lisa Nessel, Susan L. Furth, Wendy E. Hoy, Seiichi Matsuo, Gert Mayer, Shona Methven, Franz Schaefer, Elke S. Schaeffner, Laura Solá, Bénédicte Stengel, Christoph Wanner, Luxia Zhang, Adeera Levin, Kai-Uwe Eckardt, Harold I. Feldman

**Affiliations:** 1 Department of Nephrology and Hypertension, University of Erlangen-Nürnberg, Erlangen, Germany; 2 Department of Biostatistics and Epidemiology, Center for Clinical Epidemiology and Biostatistics, Perelman School of Medicine, University of Pennsylvania, Philadelphia, USA; 3 Division of Pediatric Nephrology, Children’s Hospital of Philadelphia, Philadelphia, USA; 4 Centre for Chronic Disease, School of Medicine, University of Queensland, Brisbane, Queensland Australia; 5 Department of Nephrology, Nagoya University Graduate School of Medicine, Nagoya, Japan; 6 Nephrology and Hypertension, Innsbruck Medical University, Innsbruck, Austria; 7 School of Clinical Sciences, University of Bristol, Southmead Hospital, Westbury-on-Trym, Bristol, UK; 8 Division of Pediatric Nephrology, Center for Pediatrics and Adolescent Medicine, University of Heidelberg, Heidelberg, Germany; 9 Institute of Public Health, Charite University Medicine, Berlin, Germany; 10 Directora Division Epidemiologia, DIGESA-Ministerio Salud Publica, Montevideo, Uruguay; 11 Université Paris-Saclay, Univ Paris-Sud, UVSQ, CESP, Centre for Research in Epidemiology and Population Health, Inserm, F-CRIN-INI-CRCT, Villejuif, France; 12 Department of Internal Medicine I, Division of Nephrology, University of Würzburg, Würzburg, Germany; 13 Renal Division, Department of Medicine, Peking University First Hospital, Peking University Institute of Nephrology, Beijing, China; 14 Division of Nephrology, Department of Medicine, Faculty of Medicine, University of British Columbia, Vancouver, BC Canada; 15 Department of Medicine, Perelman School of Medicine at the University of Pennsylvania, Philadelphia, USA

**Keywords:** Cohort study, Network, CKD, Epidemiology, Diversity

## Abstract

**Background:**

Chronic kidney disease (CKD) is a global health burden, yet it is still underrepresented within public health agendas in many countries. Studies focusing on the natural history of CKD are challenging to design and conduct, because of the long time-course of disease progression, a wide variation in etiologies, and a large amount of clinical variability among individuals with CKD. With the difference in health-related behaviors, healthcare delivery, genetics, and environmental exposures, this variability is greater across countries than within one locale and may not be captured effectively in a single study.

**Methods:**

Studies were invited to join the network. Prerequisites for membership included: 1) observational designs with *a priori* hypotheses and defined study objectives, patient-level information, prospective data acquisition and collection of bio-samples, all focused on predialysis CKD patients; 2) target sample sizes of 1,000 patients for adult cohorts and 300 for pediatric cohorts; and 3) minimum follow-up of three years. Participating studies were surveyed regarding design, data, and biosample resources.

**Results:**

Twelve prospective cohort studies and two registries covering 21 countries were included. Participants age ranges from >2 to >70 years at inclusion, CKD severity ranges from stage 2 to stage 5. Patient data and biosamples (not available in the registry studies) are measured yearly or biennially. Many studies included multiple ethnicities; cohort size ranges from 400 to more than 13,000 participants. Studies’ areas of emphasis all include but are not limited to renal outcomes, such as progression to ESRD and death.

**Conclusions:**

iNET-CKD (International Network of CKD cohort studies) was established, to promote collaborative research, foster exchange of expertise, and create opportunities for research training. Participating studies have many commonalities that will facilitate comparative research; however, we also observed substantial differences. The diversity we observed across studies within this network will be able to be leveraged to identify genetic, behavioral, and health services factors associated with the course of CKD. With an emerging infrastructure to facilitate interactions among the investigators of iNET-CKD and a broadly defined research agenda, we are confident that there will be great opportunity for productive collaborative investigations involving cohorts of individuals with CKD.

**Electronic supplementary material:**

The online version of this article (doi:10.1186/s12882-016-0335-2) contains supplementary material, which is available to authorized users.

## Background

Chronic kidney disease (CKD) represents a large burden of morbidity across the globe affecting between 10 and 16 % of all adults [1–6]. Importantly, CKD has been identified to substantially elevate the risk of cardiovascular disease and mortality [7]. When diagnosed early, progression of CKD can be delayed, postponing the potent negative impact of end-stage renal disease (ESRD) on quality of life and survival. Despite the size of the burden, CKD is under-acknowledged as a public health concern. While it is well recognized that CKD augments the risk for cardiovascular disease [8], it is often not mentioned in public health agendas, thus limiting research opportunities from public funding agencies.

Because the natural history of CKD is often long, CKD is challenging to study. Its etiologies are numerous and some are rare. Outcomes from CKD such as dialysis and transplantation may take years to occur, and over that time, intercurrent illness, health behaviors, and environmental exposures may alter its course, accelerate its progression, and lead to premature death from cardiovascular disease. Further, there is a large amount of clinical variability among individuals with CKD making yet more complex the design and conduct of clinical studies, with or without interventions. This variability is yet greater across national boundaries than within any one locale in light of the associated variations in health-related behaviors, healthcare delivery, genetics and environmental exposures.

An improved understanding of the natural history of CKD, of factors associated with its progression and the occurrence of morbid complications, as well as its impact on quality of life, is urgently required. This detailed understanding is essential if we are to design robust interventional studies, and potentially improve the outcomes of those living with CKD.

Well-designed cohort studies permit rigorous tracking of individual patients over time. There are numerous examples of important long-term cohort studies in cardiovascular disease (Framingham Heart Study, Nurses’ Health Study, etc.). Several large cohort studies have also collected a limited set of kidney function parameters and helped us to understand CKD prevalence and associations between CKD and cardiovascular disease. More recently, a number of cohort studies focused on CKD have been implemented in different parts of the world. These prospective studies have many similarities, and in general, aim to investigate the factors associated with progression of CKD and its negative health consequences.

The purpose of this paper is to describe the current state of these national and regional cohort studies, currently organized in a ‘virtual‘ network called iNET-CKD, to form the basis for collaborative research and research training in CKD. We describe the criteria for membership in this network, contrast the current member studies, and highlight the types of questions this network is well-positioned to address.

## Methods

### History of the network

Building on informal collaborations among the CRIC study (USA), CKD-JAC (Japan), GCKD (Germany) and C-Stride (China), members of this network participated in a 2010 NIH-sponsored “inventory meeting” of ongoing studies of CKD around the globe. A formal call for participation was organized in 2011, by investigators from CRIC and GCKD, and leveraging knowledge of other established cohorts, using some general criteria (listed below). With facilitation and endorsement by the ISN in 2012, the iNET-CKD network was formally established.

### Goals of the iNET-CKD (Fig. 1)

**Fig. 1:**
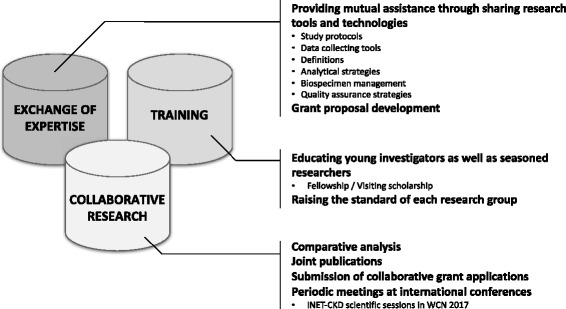
Three key goals of iNET-CKD

#### Collaborative research

Perhaps most important, the network strives to promote opportunities for joint research involving two or more network members. These collaborations include the work on common research questions, either through merging of existing data or through joint analyses of bio-samples. We also hope to increase interest in studies to validate one another’s findings. This research will permit examination of different patterns of disease around the globe and expand our understanding of the diversity of clinical manifestations of CKD.

#### Exchange of expertise

The second task of this network is to create an atmosphere where research groups can provide assistance to one another for advice and guidance concerning questions and problems related to study design and implementation. Shared information at basic levels of study design, such as data structure and variable definitions, will enhance the interpretability of analyses integrating data from multiple cohort studies. Creating an environment for sharing analytical methods and funding opportunities will benefit the cohort studies in the network and promote their ability to extend their scope through development of ancillary studies. Using web-based communication tools among members of the network, we will be able to exchange ideas and have access to a website where questions can be posted.

#### Training

This network also seeks to facilitate training opportunities for young investigators at sites with sufficient infrastructure to support such training. As well, the network promotes opportunities for experienced researchers to visit other network sights as visiting scholars and professors. Training opportunities offer sustainability and succession planning to all members, which is essential for ongoing support.

### Criteria for CKD Cohort Inclusion in the iNET-CKD

Through a consensus-building exercise, a set of common attributes have been defined as prerequisites for membership into the network. These include: 1) observational research designs with *a priori* hypotheses and defined study objectives, patient-level information, and prospective data collection including a broad spectrum of CKD-related health outcomes and bio-samples, all focused on predialysis CKD patients; 2) a minimum sample size of 1000 patients for adult cohorts and 300 for studies of children; and 3) a minimum follow-up time of three years. Not included in the network are studies: 1) that focus only on ESRD patients; 2) of the general population; and 3) that are randomized controlled trials, which may have targeted highly selected patient groups that may not represent the larger CKD patient population (Table 1). As a result, iNET-CKD primarily includes prospective observational studies of predialysis CKD patients; however, due to the first and second aims mentioned above, it also facilitates tiered membership levels for regional registries of CKD patients or well-designed retrospective cohort studies for which all inclusion criteria have not been met.

**Table 1:** Inclusion and exclusion criteria

Inclusion criteria	Exclusion criteria
• Observational studies of CKD	• ESRD cohorts
▪ Based on a priori hypotheses and study objectives	• General population cohorts
▪ Study individuals with CKD	• Randomized controlled trials
▪ Collect longitudinal data prospectively	• Clinical databases
▪ Examine a broad spectrum of CKD-related health outcomes	
▪ Collect and analyze bio-samples	
• Sample size ≥1000 in adult studies and ≥300 in pediatric studies	
• Duration of follow up ≥3 years	

### Online questionnaire

To catalog participating studies, we sent out an online survey to 15 prescreened study groups in January 2014, in addition to scanning published protocols and baseline papers of each study. The questionnaire included 121 questions about general information of the study design (e.g. size, locale, duration, goals, and inclusion/exclusion criteria), baseline demographics (e.g. age, sex, estimated glomerular filtration rate (eGFR) levels, and availability of information on socioeconomic status, comorbidities, and lifestyle risks), laboratory measurements (e.g. availability and frequency of measurements), sample collections, imaging studies, and outcome information. Data from each study’s principal investigator were obtained electronically. If needed, data were updated through publications and direct contact with the individual study groups. Each study was approved by its respective institutional review boards. No additional approval was obtained to gather summary information for this report, an activity that involved no additional collection of information from study participants.

## Results

### Participating studies

**Fig. 2:**
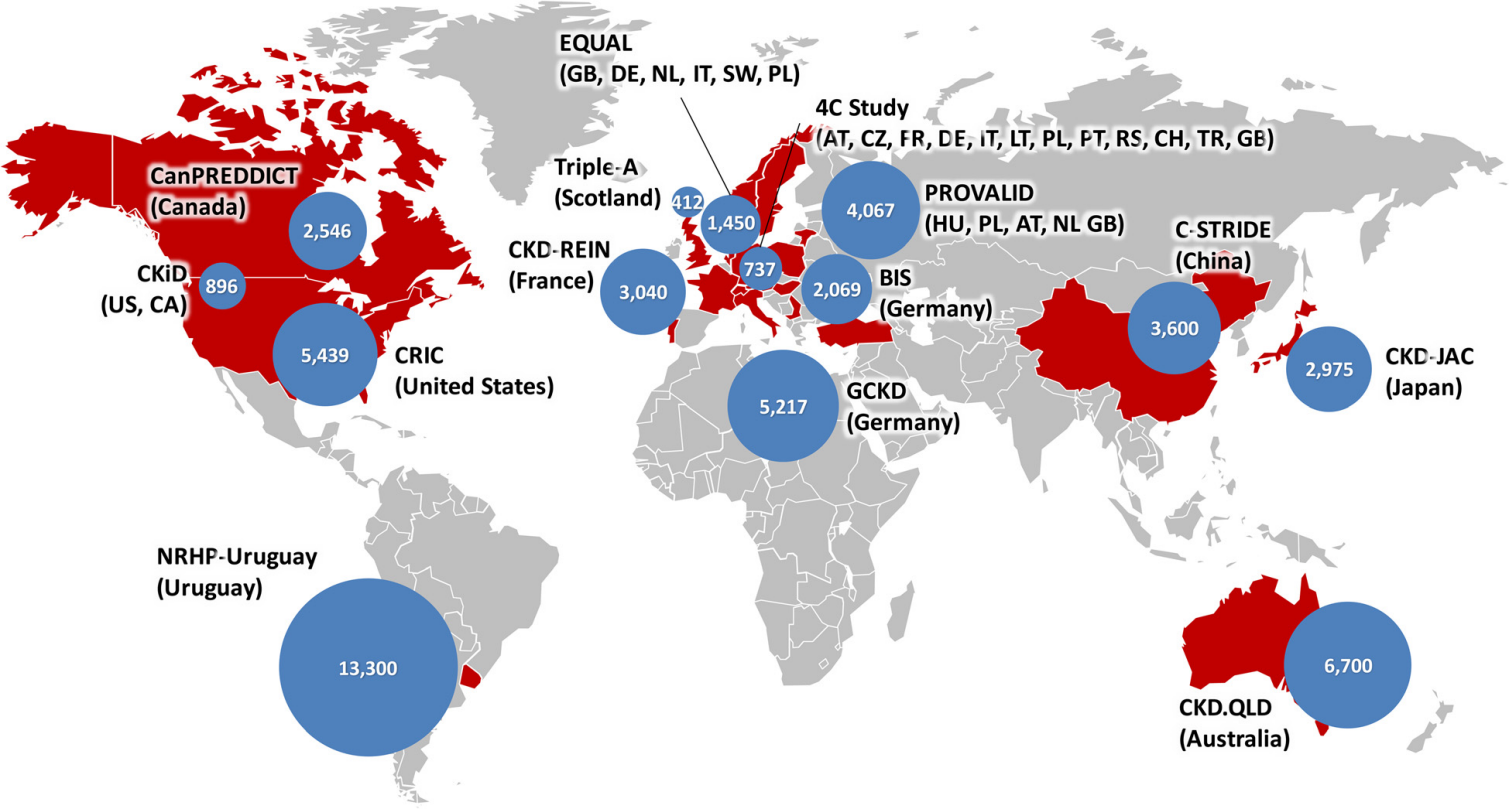
Participating studies in iNET-CKD. Countries in red represent origin of study. Blue circles represent corresponding sample size. Abbreviations: AT, Austria; CA, Canada; CH, Switzerland; CZ, Czech Republic; DE, Germany; FR, France; GB, United Kingdom; HU, Hungary; IT, Italy; LT, Lithuania; NL, Netherlands; PL, Poland; PT, Portugal; RS, Serbia; SW, Sweden; TR, Turkey; US, United States. This figure was obtained courtesy of Microsoft Office website (https://templates.office.com/en-us/Maps). No additional permission is required for its use

Principal investigators from 14 studies, including 12 cohort studies, responded to the survey and consented to participation. As a result, the network currently has enrolled 14 studies operating in 21 different countries in Asia, Europe, Australia and North America (Fig. 2). Some of these cohort studies are still enrolling; hence the exact number of study participants continues to grow. Apart from the 12 cohort studies, this network, as mentioned above, also includes large registry studies from Uruguay and Australia, where patients are entered in a database by healthcare providers who participate in the National Renal Healthcare Program (NRHP-Uruguay) and the Queensland Health Renal services, respectively. As of September 30, 2015, this network included nearly 32,000 patients from cohort studies and 20,000, from registries.

### Target populations of the participating studies

Table 2 summarizes the characteristics of the target population for each study. Some studies have very narrow eGFR inclusion ranges. However, it is obvious that this study group, as a whole, includes CKD patients with a wide range of renal function. CKiD and the 4C Study were designed exclusively for pediatric CKD patients. Both registry-type studies, the CKD.QLD and the NRHP-Uruguay, only include adult CKD patients (18 years or older). Most studies include multiple ethnicities; however, several such as BIS, CKD-JAC, GCKD, and Triple-A, only include a single ethnicity. All studies have enrolled participants from multiple centers that were, in many but not all cases, academic facilities.

**Table 2:**
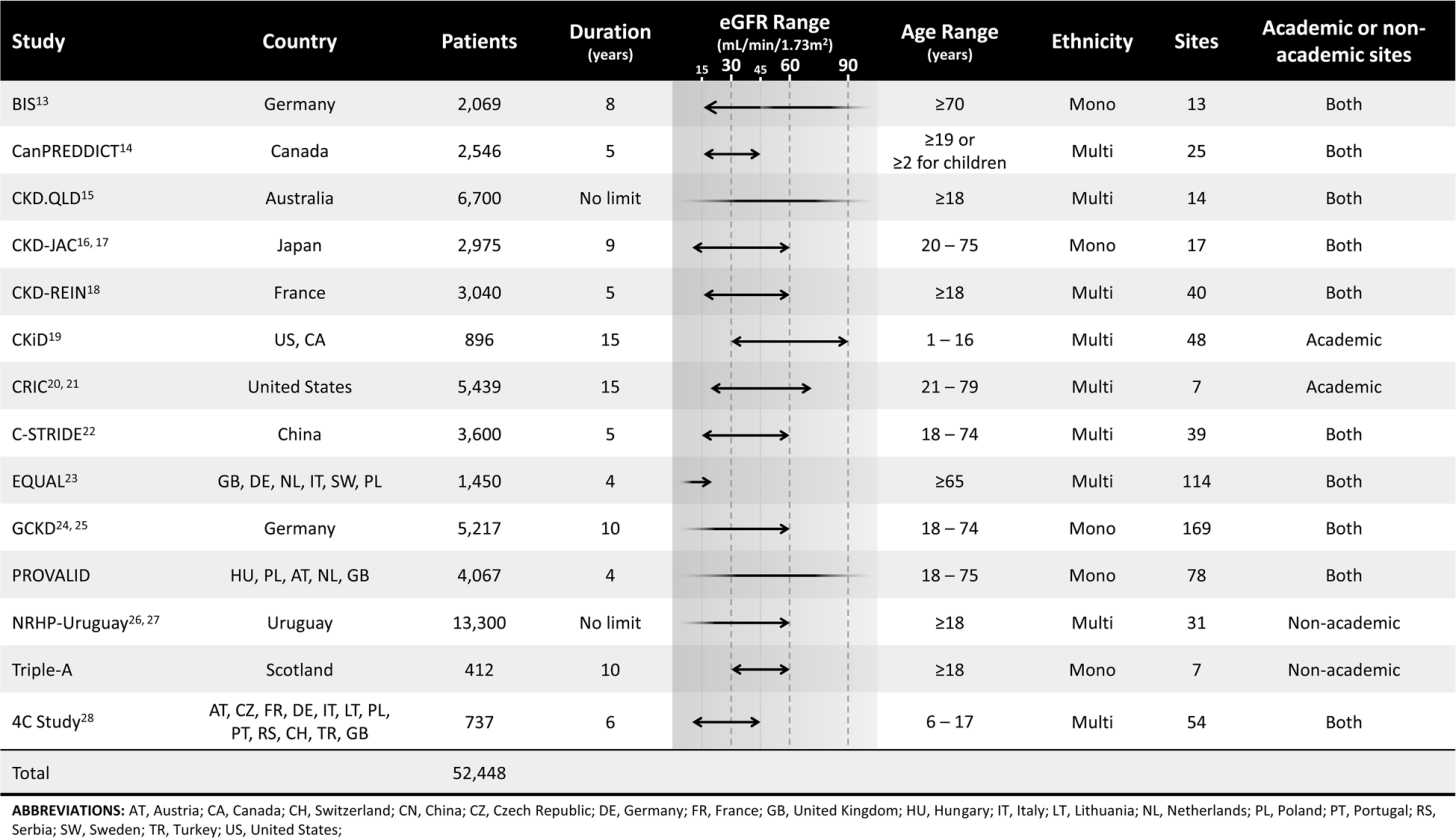
Summary table for characteristics of the target populations of participating studies in iNET-CKD

### Kidney measures, baseline covariates, and follow-up intervals

All participating studies have either biennial or annual follow-up, which includes serum creatinine and urine albumin-to-creatinine ratios or urine protein-to-creatinine ratios. Despite lack of standardization of measurements of common laboratory parameters, calibration may be achieved through exchange of samples and detailed descriptions of the collection process. All studies have data on potential confounders of associations between kidney function and outcomes. Among these are: age, race/ethnicity, smoking, history of cardiovascular disease, diabetes mellitus, hypertension status, and body mass index. Furthermore, all studies collect data on medications and all, except one, on hospitalization. Although major laboratory parameters, such as serum creatinine and albumin, are being measured annually in most studies, there is some heterogeneity in the data collection with regard to imaging studies (Table 3). Six studies have collected echocardiograms, while two and six studies have collected ankle-brachial index (ABI) and pulse wave velocity (PWV) data, respectively.

**Table 3:** Summary table for imaging studies in the participating studies

Study	ECG	Echocardio	ABI	ABPM	PWV
BIS	-	-	-	+	+
CanPREDDICT	+	+	-	-	-
CKD.QLD	-	-	-	-	-
CKD-JAC	+	+	+	+	+
CKD-REIN	-	+	+	-	-
CKiD	-	+	-	+	+
CRIC	+	+	+	+	+
C-STRIDE	+	+	-	+	+
EQUAL	+	-	-	-	-
GCKD	-	-	-	-	+
PROVALID	-	-	-	-	-
NRHP-Uruguay	-	-	-	-	-
Triple-A	+	-	-	-	-
4C Study	+	+	-	+	+

*Abbreviations*: *ECG* electrocardiogram, *Echocardio* echocardiogram, *ABI* ankle-brachial index, *ABPM* ambulatory blood pressure monitoring, *PWV* pulse wave velocity. Note that all of these imaging studies are not performed systematically

### Collection of biosamples

**Fig. 3:**
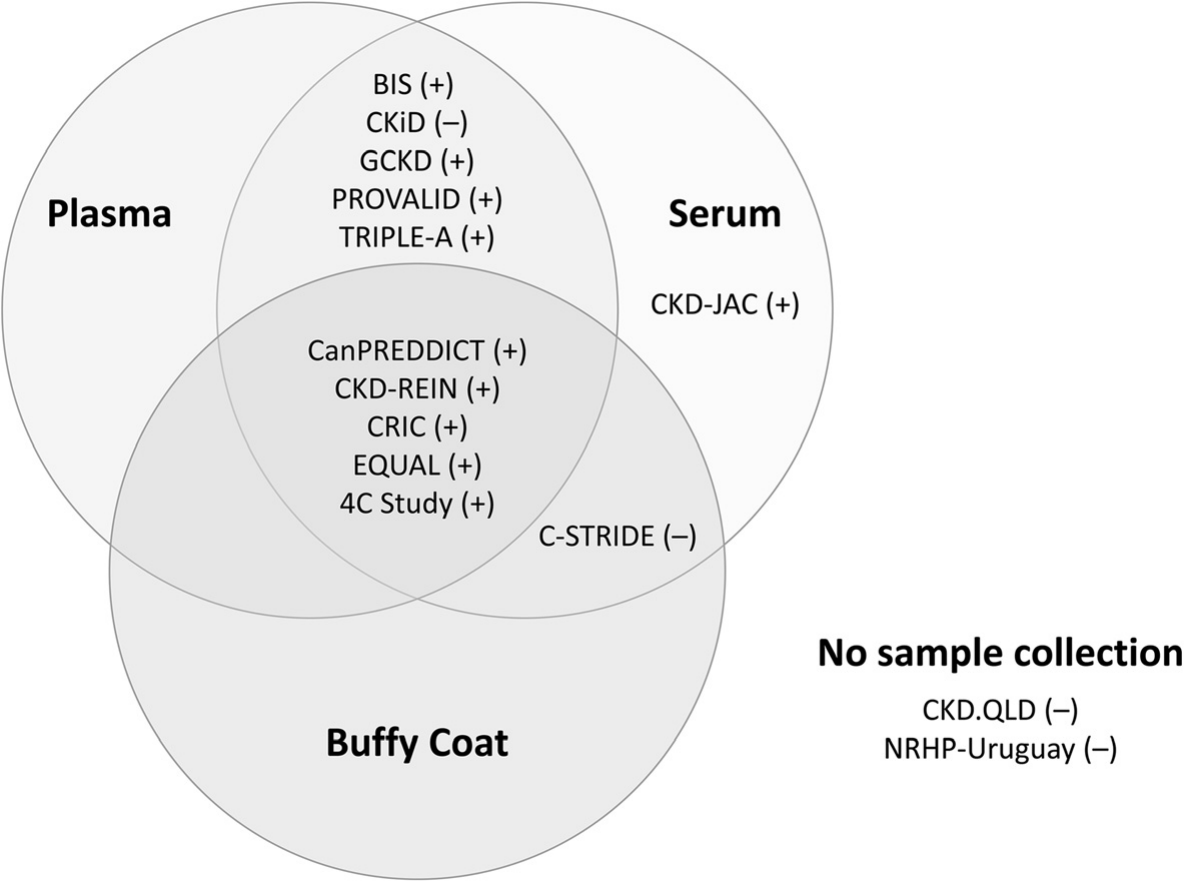
Graphical summary of blood sample collections in the participating studies. The plus or minus signs following the study names denote the availability of residual samples for further analyses

Most studies have collected bio-samples. Figure 3 summarizes blood sample collection across the studies, organized by the type of blood samples preserved and whether samples are available for further analyses for harmonization of the laboratory markers or for the measurement of novel, common biomarkers. Some studies are preserving buffy coats from which genetic information may be extracted, while other studies, such as CRIC, CKiD, BIS, and GCKD, have already collected DNA samples for genetic analyses. In CRIC and CKiD, hair and/or nails have also been collected for chemical analyses. Blood and urine sample collections have been done not only at baseline, but also at follow-up visit repeatedly in most studies (Table 4).

**Table 4:** Summary table for the presence and frequency of blood and urine samplings

Study	Blood Samples		Urine Samples	
	Baseline	Follow up	Baseline	Follow up
BIS	+	<1/year	+	<1/year
CanPREDDICT	-	≥1/year	-	≥1/year
CKD.QLD	-	-	-	-
CKD-JAC	+	≥1/year	+	-
CKD-REIN	+	<1/year	+	<1/year
CKiD	+	≥1/year	+	≥1/year
CRIC	+	≥1/year	+	≥1/year
C-STRIDE	+	≥1/year	+	≥1/year
EQUAL	+	≥1/year	+	≥1/year
GCKD	+	<1/year	+	<1/year
PROVALID	+	≥1/year	+	≥1/year
NRHP-Uruguay	-	-	-	-
Triple-A	+	-	+	-
4C Study	+	≥1/year	+	≥1/year

### Outcome variables

All studies collect information on renal outcomes, e.g. progression to ESRD (initiation of dialysis or kidney transplantation), doubling of creatinine, as well as cardiovascular events (myocardial infarction, heart failure, stroke, etc.), cardiovascular mortality and all-cause mortality (Additional file 1: Table S1). Adjudication of these outcomes, to some extent, is conducted in each of these studies, although there is not a uniform method by which this is done (Additional file 2: Table S2).

## Discussion

### Commonalities and differences

Participating studies have many commonalities that will facilitate comparative research. Nevertheless, there are also major differences. Some have very unrestricted inclusion criteria in terms of age, race, and renal function (e.g. CanPREDDICT, CKD-REIN, and CRIC). Among these, the CRIC study, however, only includes adult patients. CKiD, another US-based study, examines CKD specifically in children ages 1 through 16, which covers some of the age-range missing in the CRIC study cohort. On the other hand, BIS, a study based in Berlin, Germany, only included patients aged ≥70 years. Principally due to relatively homogenous populations, some studies from Europe and Asia include only Caucasians (e.g. GCKD) or Asians (e.g. CKD-JAC or C-STRIDE), while others are ethnically diverse (e.g. CRIC).

Furthermore, some studies focus on more severe stages of renal disease. The EQUAL study, based in six European countries, includes patients with an eGFR of ≤20 ml/min who are 65 years or older. PROVALID, another multi-country study based in Europe, is limited to investigating renal endpoints in subjects with type 2 diabetes.

### Structure

iNET-CKD is an open network. Central coordination is supported by the ISN, which provides administrative services and facilitates linkages with regional boards and programs, including research programs, training opportunities amongst others (http://www.theisn.org/initiatives/inet-ckd). As an international organization committed to linking the developed and developing world, and to excellence in science and education, the ISN is uniquely positioned to facilitate this initiative, while the leadership of the group maintains the scientific autonomy for goal-setting within the group of iNET-CKD participating studies.

### Specific features of iNET-CKD as compared with other CKD consortia

The creation of this iNET-CKD will provide a unique opportunity for a scientific exchange among CKD investigators around the globe, as well as to enhance training opportunities for young researchers. The diversity represented within the network will facilitate the identification of yet-unknown factors (genetic, health-related behaviors, healthcare delivery) associated with the development and the course of CKD. Such findings may not only have implications for clinical care of patients with CKD but also for health policy makers.

The prospective design of the participating studies is associated with a high quality of study data and the potential to combine study samples across the network will enhance the ability to examine multiple health outcomes related to CKD.

iNET-CKD is not the only network with the goal to advance research in CKD; there are a number of disease-specific cohort studies and consortia, such as NEPTUNE [9] and INSIGHT [10] for nephrotic syndrome and collaborative studies on genetic epidemiology in IgA nephropathy [11], CKD (CKDGen [12]), and pediatric nephrology [13]. However, few studies include a wide range of CKD patients. The Chronic Kidney Disease Prognosis Consortium (CKD-PC), established in 2009, is a network of investigators who have access to a minimum set of data from about fifty cohorts or clinical trial populations from around the world drawn from the general-population, populations at high-risk for kidney disease, and populations with CKD [14]. The ability to process information from a diverse set of data, using common analytic plans and codes has proven to be a valuable asset in CKD research studying the prognostic impact of eGFR and albuminuria, as well as establishing the staging of CKD and helping to revise practice guidelines [15]. Complementary to the CKD-PC, iNET-CKD primarily focuses on CKD cohort studies, involving patients with well-phenotyped CKD using extensive patient-level data,and biosamples. Rather than implementing meta-analyses, we seek opportunities to combine primary data from different groups to implement joint analyses. Moreover, iNET-CKD focuses on diverse CKD-related outcomes well beyond progression of CKD and mortality. The granularity of the collected data is very high, and biomaterials are available in almost all participating cohorts, which present the opportunity for coordinated analysis of biomarkers, and serve as an important resource for comparative studies and validation of findings. Projects in the near future involve observations on international variations in dietary patterns related to dietary constituents such as phosphate and sodium as well as variations in clinical strategies for the management of CKD and their respective effects on variations in CKD outcomes.

### Strengths and limitations

The variation in inclusion criteria is one of the major strengths of this network. The studied populations are genetically distinct, which will give insight on possible genetic determinants of CKD. Furthermore, these populations also greatly differ with respect to health behaviors, healthcare delivery, and environments. For example, different utilization of medications may play a role in the rapidity of progression of CKD in some studies. This network will provide the opportunity to examine the same ethnicities in multiple countries, providing insights into the specific role of health behaviors and healthcare delivery in CKD outcomes.

This network of cohort studies has certain limitations. While commonalities in study design will facilitate joint projects, inconsistencies in the definition and capture of variables as well as adjudication of outcomes can complicate analyses. Second, races and ethnicities, other than Blacks, Whites and Asians, are underrepresented in our cohorts. Almost all Blacks are African American, and come from North American cohorts. Asians are predominantly Eastern Asian in C-Stride and CKD-JAC, while in EQUAL, South Asians; however, the sample size of South Asians may not be sufficient for stratified analyses. We, therefore, hope to include emerging studies from India and the African continent in the future.

## Conclusion

In summary, this network will aid joint research in the field of CKD around the globe. With an emerging infrastructure to facilitate interactions among investigators, the commitment of currently involved investigators to ensure responsible use of the data, and a broadly defined research agenda, we are confident that there will be ongoing development of new cohort studies around the globe that will join iNET-CKD. This international network will be in an exceptional position to validate findings across geographical and national boundaries, to test hypotheses and to generate new understanding of CKD progression and its complications. These, in turn, can be used to inform clinical trials, potentially serve as a source of patients for clinical trials, and help to inform health policy.

## Additional files

Additional file 1: Table S1. Summary of study designs and primary outcomes of interest. Provides an overview of the study designs and primary outcomes of interest for each iNET-CKD Study. Abbreviations: CKD, chronic kidney disease; ESRD, end-stage renal disease; CVD, cardiovascular disease; PWV, pulse wave velocity. (TIF 635 kb) Additional file 2: Table S2. Summary of baseline covariates, measurements, and event ascertainment among participating studies. Provides a summary of baseline covariates, study measurements, and event ascertainment activities for each iNET-CKD study. Abbreviations: IDMS Cr, IDMS-calibrated serum creatinine; ACR, urinary albumin-to-creatinine ratio; PCR, urinary protein-to-creatinine ratio; CVD, cardiovascular disease; BMI, body mass index. (TIF 1106 kb)

## Abbreviations

ABI: Ankle-brachial index
ABPM: Ambulatory blood pressure monitoring
AT: Austria
CA: Canada
CH: Switzerland
CKD: Chronic kidney disease
CZ: Czech Republic
DE: Germany
ECG: electrocardiogram
Echocardio: echocardiogram
eGFR: estimated glomerular filtration rate
ESRD: End-stage renal disease
FR: France
GB: United Kingdom
HU: Hungary
IT: Italy
LT: Lithuania
NL: Netherlands
PL: Poland
PT: Portugal
PWV: Pulse wave velocity
RS: Serbia
SW: Sweden
TR: Turkey
US: United States
