# Supplementary figures and images for: International Network of Chronic Kidney Disease cohort studies (iNET-CKD): a global network of chronic kidney disease cohorts
Source: BMC Nephrol. 2016 Sep 2;17(1):121. doi: 10.1186/s12882-016-0335-2 (PMC5010740; doi:10.1186/s12882-016-0335-2)

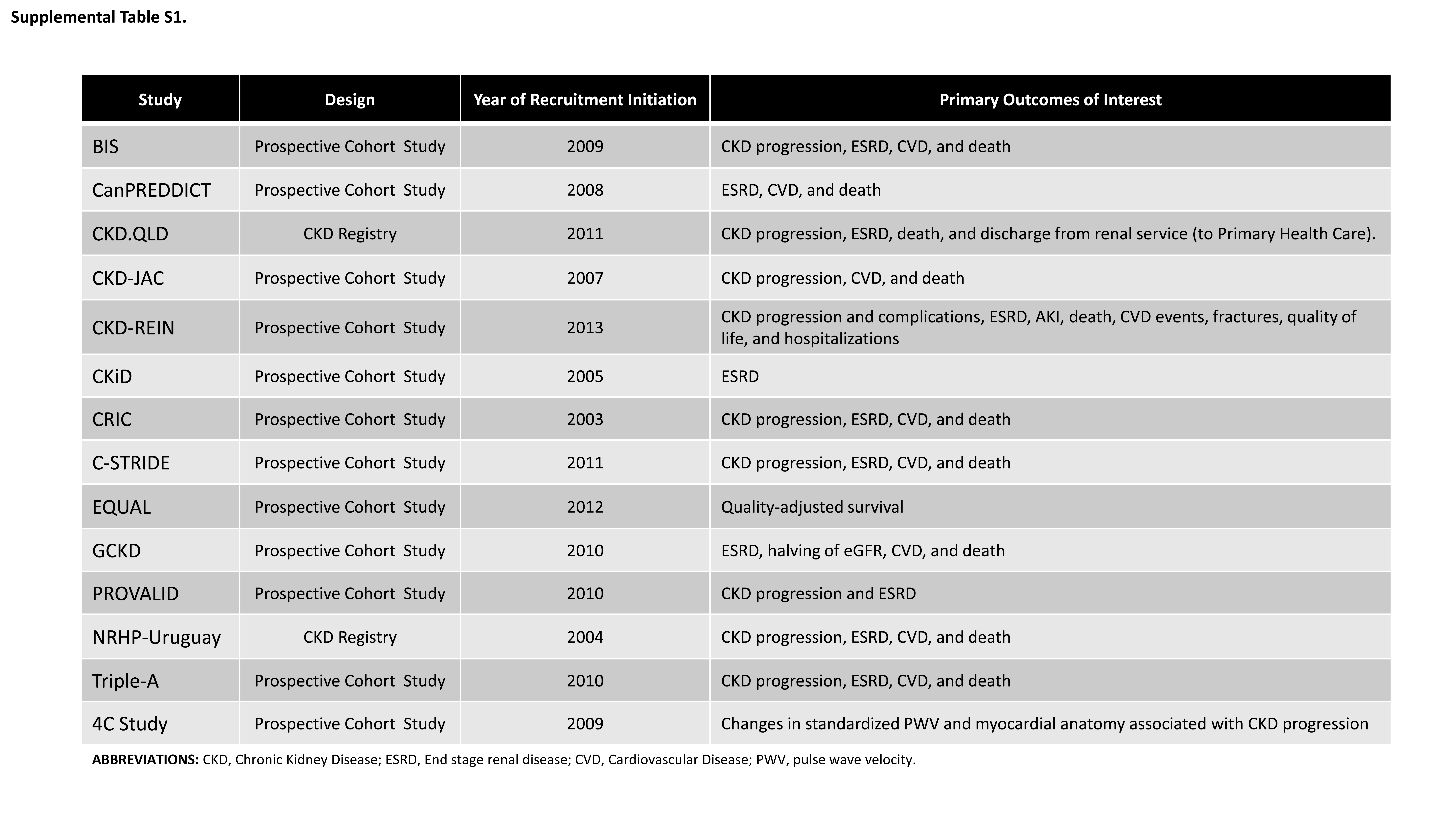

Supplement: Additional file 1: Table S1. — Summary of study designs and primary outcomes of interest. Provides an overview of the study designs and primary outcomes of interest for each iNET-CKD Study. Abbreviations: CKD, chronic kidney disease; ESRD, end-stage renal disease; CVD, cardiovascular disease; PWV, pulse wave velocity. (TIF 635 kb) [file 12882_2016_335_MOESM1_ESM.tif]

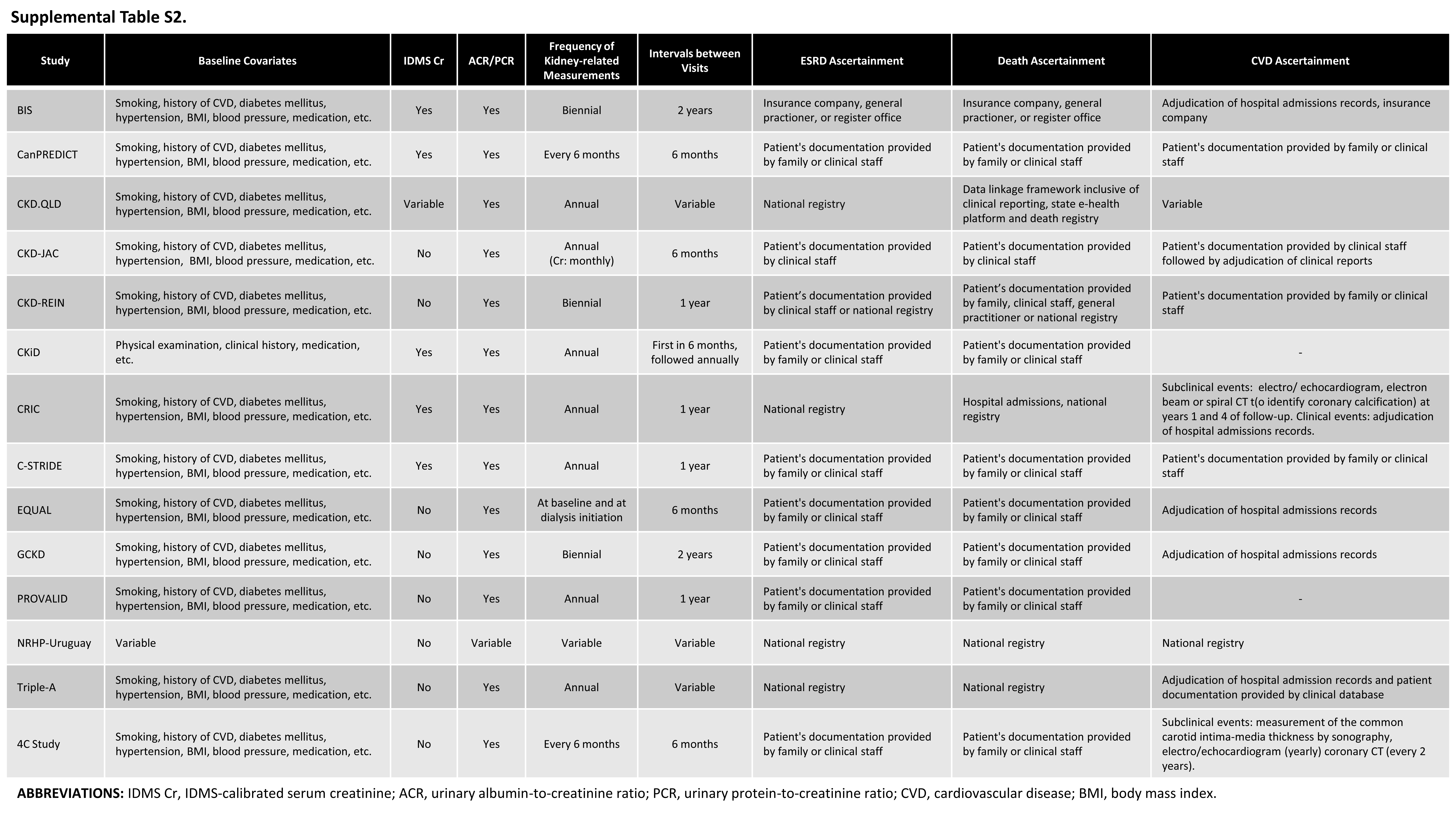

Supplement: Additional file 2: Table S2. — Summary of baseline covariates, measurements, and event ascertainment among participating studies. Provides a summary of baseline covariates, study measurements, and event ascertainment activities for each iNET-CKD study. Abbreviations: IDMS Cr, IDMS-calibrated serum creatinine; ACR, urinary albumin-to-creatinine ratio; PCR, urinary protein-to-creatinine ratio; CVD, cardiovascular disease; BMI, body mass index. (TIF 1106 kb) [file 12882_2016_335_MOESM2_ESM.tif]
